# Trypanosomatid Infections: How Do Parasites and Their Excreted–Secreted Factors Modulate the Inducible Metabolism of l-Arginine in Macrophages?

**DOI:** 10.3389/fimmu.2018.00778

**Published:** 2018-04-20

**Authors:** Philippe Holzmuller, Anne Geiger, Romaric Nzoumbou-Boko, Joana Pissarra, Sarra Hamrouni, Valérie Rodrigues, Frédéric-Antoine Dauchy, Jean-Loup Lemesre, Philippe Vincendeau, Rachel Bras-Gonçalves

**Affiliations:** ^1^CIRAD, Montpellier, France; ^2^UMR 117 ASTRE “Animal, Santé, Territoire, Risques et Ecosystèmes”, Univ. Montpellier (I-MUSE), CIRAD, INRA, Montpellier, France; ^3^UMR 177 INTERTRYP “Interactions Hôte-Vecteur-Parasite-Environnement dans les maladies tropicales négligées dues aux Trypanosomatidae”, Univ. Montpellier (I-MUSE), CIRAD, IRD, Univ. Bordeaux 2, Univ. Lyon 1, Montpellier, France; ^4^Univ. Bordeaux, UMR 177 INTERTRYP, Bordeaux, France; ^5^CHU Bordeaux, Laboratoire de Parasitologie-Mycologie, Bordeaux, France; ^6^CHU Bordeaux, Département des Maladies Infectieuses et Tropicales, Bordeaux, France

**Keywords:** macrophage activation, l-arginine metabolism, arginase, secretome, trypanosomatids

## Abstract

Mononuclear phagocytes (monocytes, dendritic cells, and macrophages) are among the first host cells to face intra- and extracellular protozoan parasites such as trypanosomatids, and significant expansion of macrophages has been observed in infected hosts. They play essential roles in the outcome of infections caused by trypanosomatids, as they can not only exert a powerful antimicrobial activity but also promote parasite proliferation. These varied functions, linked to their phenotypic and metabolic plasticity, are exerted *via* distinct activation states, in which l-arginine metabolism plays a pivotal role. Depending on the environmental factors and immune response elements, l-arginine metabolites contribute to parasite elimination, mainly through nitric oxide (NO) synthesis, or to parasite proliferation, through l-ornithine and polyamine production. To survive and adapt to their hosts, parasites such as trypanosomatids developed mechanisms of interaction to modulate macrophage activation in their favor, by manipulating several cellular metabolic pathways. Recent reports emphasize that some excreted–secreted (ES) molecules from parasites and sugar-binding host receptors play a major role in this dialog, particularly in the modulation of the macrophage’s inducible l-arginine metabolism. Preventing l-arginine dysregulation by drugs or by immunization against trypanosomatid ES molecules or by blocking partner host molecules may control early infection and is a promising way to tackle neglected diseases including Chagas disease, leishmaniases, and African trypanosomiases. The present review summarizes recent knowledge on trypanosomatids and their ES factors with regard to their influence on macrophage activation pathways, mainly the NO synthase/arginase balance. The review ends with prospects for the use of biological knowledge to develop new strategies of interference in the infectious processes used by trypanosomatids, in particular for the development of vaccines or immunotherapeutic approaches.

## Trypanosomatid Infectious Diseases and Macrophage Activation Pathways

### Infections Caused by Trypanosomatids

Trypanosomes and *Leishmania* parasites cause important but neglected infectious diseases in both humans and animals worldwide.

Sleeping sickness or human African trypanosomiasis (HAT) is an endemic parasitic disease exclusively located in intertropical Africa, caused by *Trypanosoma brucei gambiense* (*Tbg*, 95% of cases) and *T. b. rhodesiense*, transmitted by the tsetse fly (1). Related parasites, including *T. b. brucei* (*Tbb*), *T. congolense, T. vivax, T. evansi*, and *T. equiperdum*, cause wasting diseases in livestock, termed animal African trypanosomosis (AAT) or nagana, and are the cause of a few atypical cases in humans (2). HAT is a severe burden for poor rural populations (3, 4). The real number of infected people is most probably underestimated as published maps are the result of mathematical extrapolation of data recorded in only partial epidemiological surveys, a situation aggravated by wars and social conflicts (5–7). After a painful tsetse bite, the first clinical sign of HAT is the chancre at the bite site, which disappears within 2 or 3 weeks (1). The disease evolves in two distinct pathological stages. Within a few days after the tsetse bite, the patient enters the stage I also called hemolymphatic stage. Intermittent fever develops because of the successive waves of parasite replication in the blood. Adenopathies, splenomegaly, or even hepatological signs are frequent. The stage II or meningoencephalitis stage emerges slowly and insidiously over a period of months or years when the parasites invade the nervous system. The general signs of the hemolymphatic stage do not completely disappear, and the neurological symptoms develop progressively in parallel. A wide variety of neurological symptoms are encountered. The main symptoms after which sleeping sickness was named are daytime somnolence and nocturnal insomnia. Staging relies on white cell counts and detection of trypanosomes in the cerebrospinal fluid and is indispensable as the treatment of stages I and II differs (8), and it was recently demonstrated that the parasite could persist in adipose tissue (9).

American trypanosomiasis, named Chagas disease in recognition of Carlos Chagas, who first discovered it in 1909, is mostly encountered in South and Central America. Infection primarily affects poor rural populations in Latin America and has serious consequences for public health. The disease develops following infection by the protozoan parasite *Trypanosoma cruzi*. The parasite is mainly transmitted by the Triatomine vector also known as the “kissing bug” (10). The disease has two clinical stages. The initial acute stage lasts for about 2 months after infection, parasites circulate in the blood, but in most cases symptoms are mild or even absent. In less than 50% of people bitten by a bug, early characteristic clinical signs can be observed, such as skin lesion (chagoma) or unilateral edema in the eyelid (the sign of Romaña). *T. cruzi* proliferates actively in the infected individual and invades many types of host cell. The host immune response leads to a dramatic reduction in the parasite load. People then enter the chronic stage of the disease and remain asymptomatic for years. The patient shows evidence of immunity (antibodies to specific antigens of *T. cruzi*) but remains infected, and the immune system does not prevent disease progression to the chronic stage. Up to 30% of patients suffer from cardiac disorders and up to 10% suffer from digestive (typically enlargement of the esophagus or colon), neurological, or mixed alterations (11). In later years, the infection can cause sudden death due to cardiac arrhythmias or progressive heart failure caused by the destruction of the heart muscle and its nervous system. Chagas disease can also be reactivated if patients in the chronic phase are immune compromised as in the case of coinfection with HIV or due to chemotherapy (10).

Leishmaniases are vector-borne neglected tropical diseases caused by different species of the *Leishmania* protozoan parasite. They represent a major public health problem worldwide, as they are present in 98 endemic countries. Besides humans, several mammals, often domesticated or wild canids, provide an additional zoonotic reservoir of infection, especially of *L. infantum* (12). Although most people infected by *Leishmania* sp. develop no symptoms, the clinical features include a wide range of symptoms depending on the species of *Leishmania* concerned and the immune response of each host. Cutaneous leishmaniasis, the most common form of the disease causes skin sores on the exposed parts of the body (13). The sores may start out as papules or nodules and end up as ulcers with a raised edge. When the ulcers heal, they leave permanent scars, often the cause of serious social stigma. In mucocutaneous leishmaniasis, the parasite spreads from the skin and causes ulcers in the mucous membranes of the nose (most common location), mouth, or throat, which can lead to partial or total tissue destruction (14). Visceral leishmaniasis, also known as kala-azar, is characterized by irregular fever, weight loss, anemia, and enlargement of the spleen and liver and results in death if untreated (12). Severe pancytopenia is observed, and parasites are found in bone marrow. HIV/AIDS patients are much more likely to develop VL, and once infected, VL accelerates AIDS (15).

Recent investigations report an increase in arginase activity in trypanosomatid-infected patients (16–19), and for instance, arginase activity is considerably higher in the blood of VL/HIV coinfected patients than in VL patients (20) or its age-related alteration impacts on disease severity (21).

### Excreted–Secreted (ES) Factors of Trypanosomatids and Macrophage Targeting

The excretory–secretory component is the primary interface between the parasite and its host and induces strong molecular crosstalk with its environment. Studies of naturally ES factors by microorganisms have dramatically increased in recent years, including viruses, bacteria, and parasites. The ES factors or secretome of trypanosomatids is a complex mixture of proteins, carbohydrates, and lipids excreted from the surface of the parasite or secreted through the flagellar pocket of the parasite and *via* exocytosis vesicles (22). The composition of this complex mixture is still largely unknown, despite the recent definition of an experimental approach for the identification of conserved secreted proteins in trypanosomatids (23), but it has long been suspected of being important for the parasitic lifestyle (24).

For example, the whole secretome of *Tbg* was shown to be able to inhibit the maturation of dendritic cells (DCs) and the induction of lymphocytic allogenic responses (25). In addition, there is evidence for the involvement of diverse enzyme families, such as proteases and hydrolases, in different aspects of the pathogenesis in human hosts (26–28). Regarding HAT, the whole secretome of three different bloodstream strains of *Tbg* were analyzed using a proteomics-based approach, which enabled the identification of over 440 proteins, several of which were described for the first time (29). Moreover, the secretome molecular profile was associated with the virulence of the parasite *in vivo* (30). Similar studies were conducted in species responsible for animal trypanosomoses, particularly *T. congolense* and *T. evansi* (31, 32). They evidenced a core secretome and specificities in African trypanosomes affecting humans and those affecting animals. In addition, results obtained with *Tbg* were compared with both the glycosome and with the total proteome of a *Tbb* strain, highlighting the importance of protein isoforms between the parasite cellular metabolism and its corresponding ES molecules (29). Interestingly, a large proportion of the secreted proteins were found in vesicles displaying active exocytosis beyond the flagellar pocket. Trypanosomes of the *brucei* group produce nanotubes coming from the flagelle, which dissociate into vesicles. Vesicles from *T. b. rhodesiense* contain the serum resistance-associated protein, which can be transferred to *Tbb* leading to evasion to human innate immunity (33, 34). This new type of secretion could be crucial for the survival strategy of *Trypanosoma* by allowing them to exchange proteins at least between parasites and/or to manipulate the host immune system.

For *T. cruzi*, it was also evidenced that proteins are released *via* vesicles formed by at least two different mechanisms, larger ectosomes budding from the plasma membrane and smaller exosomes within the flagellar pocket (35). Proteomics enabled the identification of proteins involved in metabolism, signaling, nucleic acid binding, and parasite survival and virulence. The authors concluded that *T. cruzi* uses different secretion pathways to excrete/secrete proteins and that infective forms of the parasite may use the extracellular vesicles to deliver cargo to the host cells (35). A recent comparative proteomic analysis demonstrated both common and specific proteins in the secretomes from two different *T. cruzi* strains, highlighting, similar to African trypanosomes, a plasticity probably associated with the parasite virulence (36).

Exosome-like microvesicles were also evidenced in *L. donovani*. Proteomics revealed a large majority of known eukaryotic exosomal proteins in the conditioned medium of cultured parasites (37). These proteomics results were extended to *L. braziliensis*, for which only 5% of the identified secreted proteins presented a classical secretion signal (38). Interestingly, these exosome-like vesicles were further shown to be involved in the communication with macrophages and immune modulation (39) and could be involved in immune evasion (40). Of importance, *Leishmania* exosomes presented mainly pro-parasitic activities, both *in vitro* and *in vivo*, functionally priming host cells in the first moments of the infection (41) or in the establishment of the disease (42). Moreover, *L. infantum* secretes various molecules that modulate human DC differentiation and functions (43).

**Figure 1:**
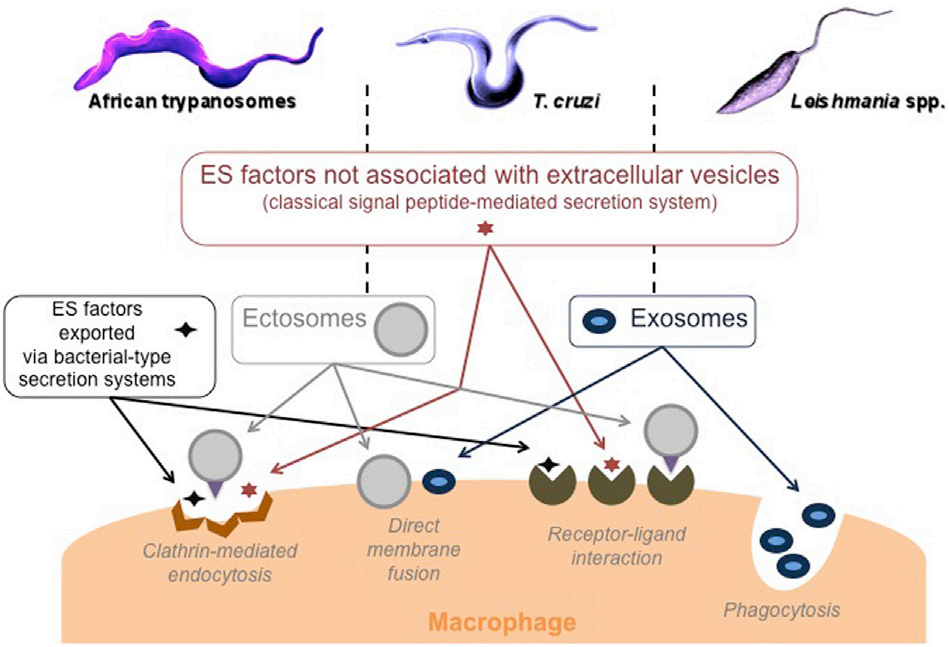
Trypanosomatid modes of secretion and macrophage targeting. ES, excreted–secreted, ectosomes: ubiquitous microvesicles assembled at and released from the plasma membrane, and exosomes: nanovesicles released on the exocytosis of multivesicular bodies.

Among the functional classes of ES factors, the group of unfolding and degradation proteins, mainly proteases, deserves the most attention. They cover a large panel of physiological and pathological functions, and representatives of this group are known to be virulence factors, to favor parasite invasion and its growth in the hostile host environment, to make it possible to escape the host immune defenses, and/or, finally, to produce nutrients by hydrolyzing host proteins (44–47). In addition, trypanosomatids can use at least four secretory systems to sequentially deliver factors to modulate macrophage response and consequently the response of the immune system as a whole; the classical signal peptide-mediated system as well as bacterial-type secretion systems that export proteins directly into the host environment, and two vesicular systems, including ectosomes and exosomes. These extracellular vesicles are specifically released by trypanosomatids to deliver signals to the target cells. Aside from considerable differences in content and morphology, with some ubiquitously assembled and released from the plasma membrane while others are released during exocytosis of the multivesicular bodies, the functions of ectosomes are largely analogous to those of exosomes (48). The study of these extracellular vesicles and their importance in biological communication is in full swing (49, 50), even using a philosophical approach (51), which could be appropriate in the case of parasites such as trypanosomatids (33, 34, 52). Interestingly, the different modes of secretion can also interact in different ways with the macrophage: *via* receptor–ligand interactions (free proteins and ectosomes), endocytosis (free proteins and ectosomes), phagocytosis (exosomes), or by direct fusion with the plasma membrane (Figure 1).

### Macrophage Activation Pathways

#### Classical Versus Alternative Activation Pathway

**Figure 2:**
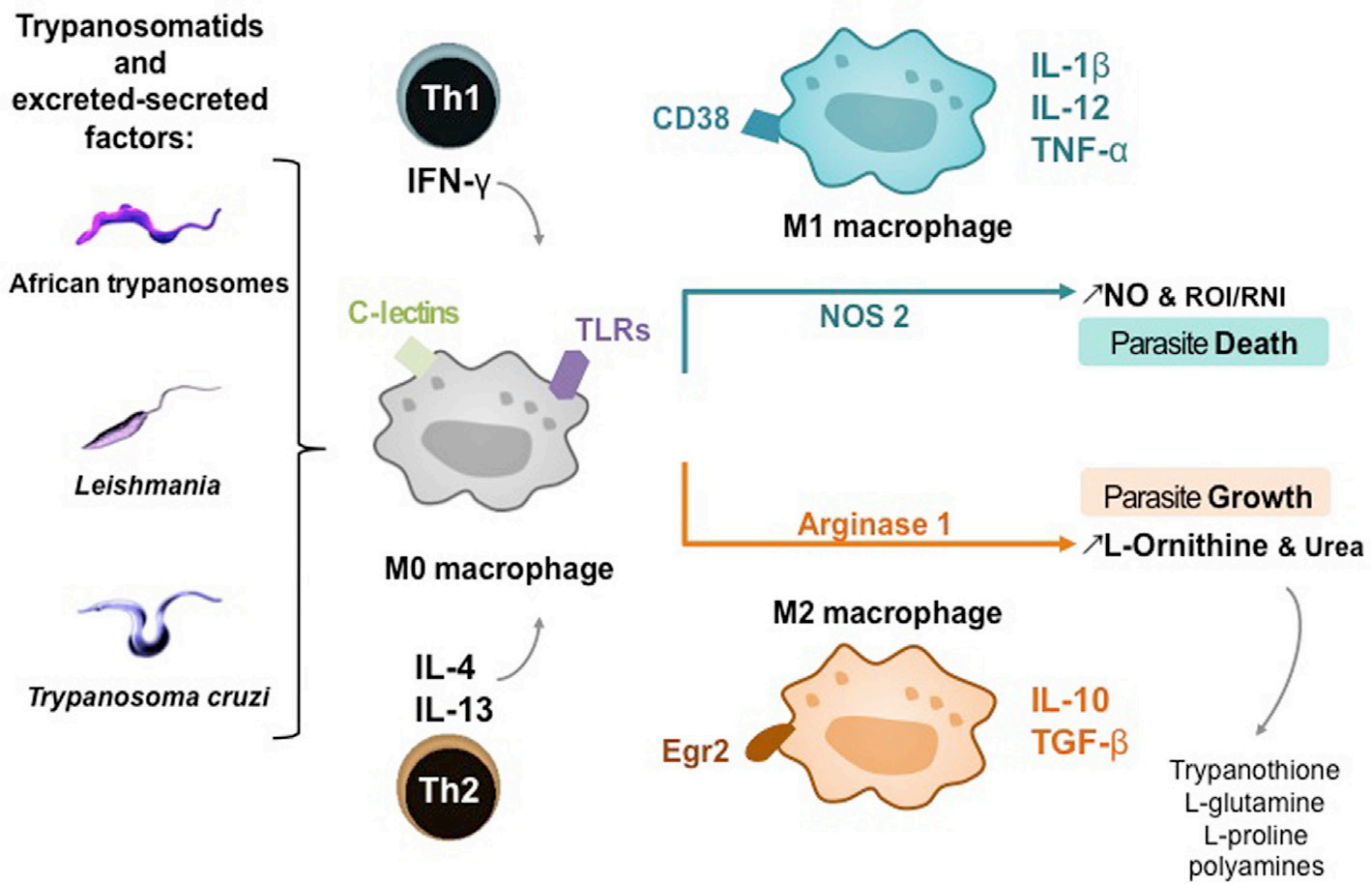
Macrophage activation pathways in trypanosomatid infections. T-cell subsets potentiating M0 macrophage differentiation into M1 or M2 subtype; Th, helper T lymphocyte; IFN-γ, interferon-γ; IL, interleukin. Host cell receptors involved in trypanosomatid detection: C-lectins and toll-like receptors (TLRs). Phenotypic markers and cytokines of macrophage polarization: cluster of differentiation (CD) 38, Egr2, early growth response protein 2; TNF-α, tumor necrosis factor; TGF-β, transforming growth factor. Products of macrophage polarization influencing the death or growth of trypanosomatids; NO, nitric oxide.

**Figure 3:**
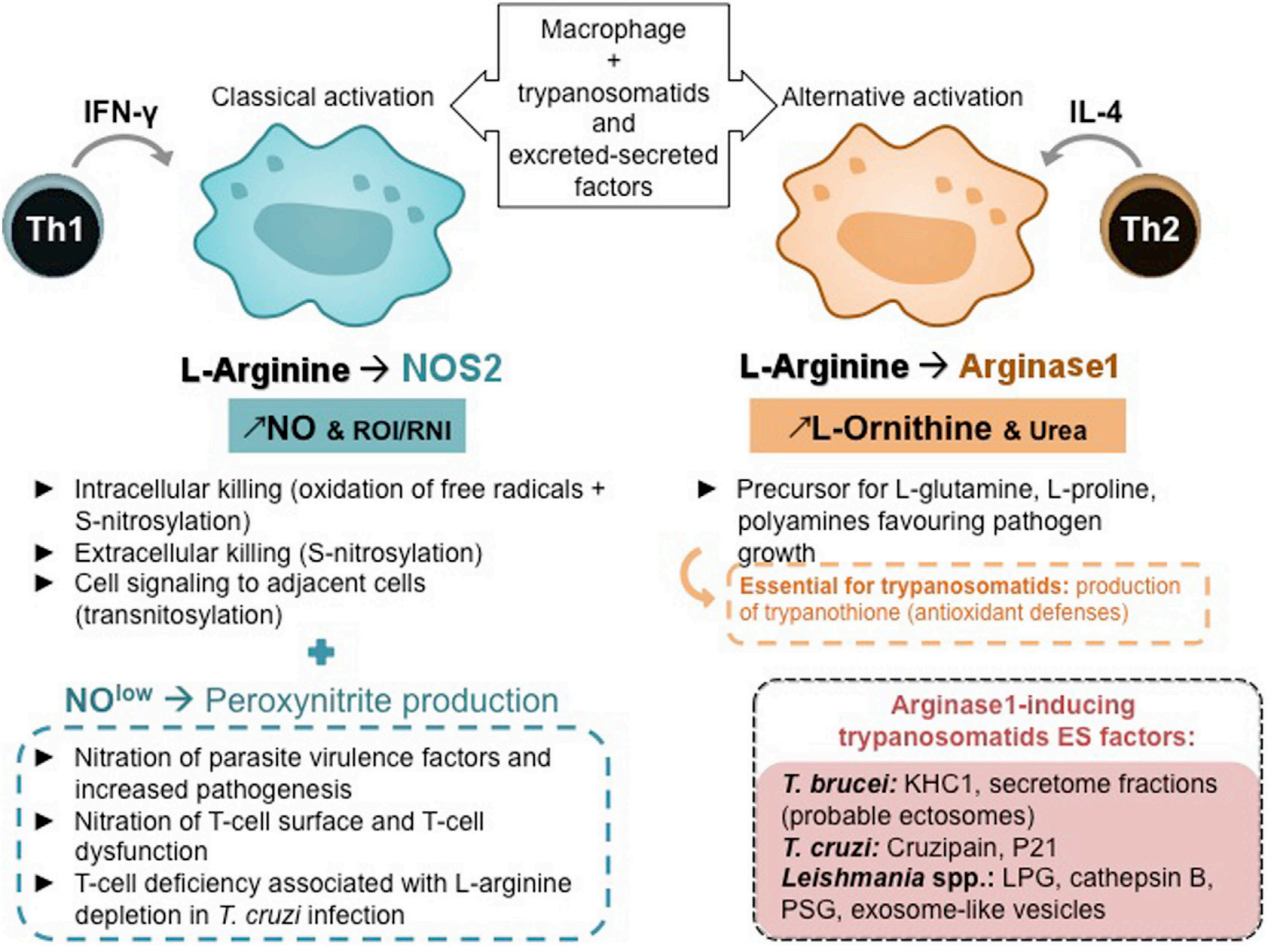
Macrophage polarization, T-helper subsets, and l-arginine metabolism. Th, helper T lymphocyte; IFN-γ, interferon; IL, interleukin; NO, nitric oxide; ROI, reactive oxygen species; RNI, reactive nitrogen species.

The main function of macrophages is to react to external stimuli, including pathogens and particularly their ES factors, to inform the host’s immune system, and to modulate the corresponding response. The functional properties of macrophages make it possible to distinguish different phenotypes of subpopulations (53). Depending on the type of cell, the cytokines and pathogens present at the infection site, unpolarized macrophages (M0) can differentiate into classically activated M1 macrophages or alternative activated M2 macrophages. The two macrophage subpopulations express different surface receptors and produce specific sets of cytokines or chemokines (54, 55). M1 are potent pro-inflammatory cells, with high microbicidal activity [e.g., expression of antigen presentation molecules (MHC II) and co-stimulation molecules (CD40 and CD80/86), secretion of tumor necrosis factor (TNF)-α, interleukin (IL)-12, and activation of nitric oxide synthase (NOS) 2], while M2, which have moderate anti-inflammatory properties (e.g., secretion of IL-10 and high levels of arginase-1), are poorly microbicidal and are involved in tissue repair (56, 57). Although it is difficult to find specific phenotypical markers to delineate M0, M1, and M2 macrophages, recent findings in mouse provide evidence that some surface markers can be considered as representative of each subtype of macrophage, such as CD38 for M1 and early growth response protein 2 for M2 (58). Taken together, the cytokine pathways, nitric oxide (NO) and polyamine levels, may explain why there is more than a simple duality of microbicidal/pro-inflammatory properties versus cell growth/anti-inflammatory properties in the macrophage subpopulations (59, 60). Actually, M1 and M2 phenotypes often coexist, and other terms have emerged to identify non-classical activation phenotypes such as M2a or M2b, the latter representing alternative activated macrophages that express small amounts of arginase 1 (56). The resulting mixed phenotype then depends on the balance between activator and inhibitor activities and the tissue environment, thereby determining the outcome of the infection (61, 62), particularly during trypanosomatid infections (Figure 2). Thus, the role of macrophage activation stimuli needs to be considered in the dynamic complexity driven by trypanosomatid parasites and particularly their ES factors (Figures 2 and 3), as well as a function of the host (63).

#### l-Arginine Metabolism Balance and T-Helper Subsets

Mammalian arginine metabolism is complex as this semi-essential amino acid is a substrate for many enzymes that may compete with each other (64). The dual role of l-arginine metabolism, its regulation by T cells, and alterations of l-arginine metabolism by pathogens were recently reviewed (65). Parasites and particularly *Leishmania* and trypanosomes are highly sensitive to the l-arginine-NO pathway (Figure 3). For instance, *L. major* infection in mice established the paradigm of Th1 and Th2 subset roles (66), a Th1 response being associated with IFN-γ production and NOS 2 expression, whereas a Th2 response being associated with IL-4 production and arginase 1 expression (67, 68). Interestingly, it has been demonstrated that a deprivation in l-arginine impairs *L. major*-specific T-cell responses (69). T-cell deficiency associated with l-arginine depletion has been evidenced in cancer and in infectious diseases including *T. cruzi* infection. A decrease in cyclin-dependent kinases essential for the cell cycle, the downregulation of T-cell receptor z chain, has been shown to be implicated in T-cell anergy (70).

The low levels of l-arginine and NO in macrophages lead to various RNI, such as peroxynitrites that can not only diffuse around macrophages and kill extracellular infectious agents and intracellular pathogens in adjacent cells but also induce the nitration of various proteins and are involved in the pathogenesis of various infections including leishmaniasis and trypanosomiasis (71–73). Products of NOS and NAPH oxidase in classically activated macrophages can react, leading to S-nitrosylation in protein resulting in the death of extracellular parasites and through transnitrosylation can affect various targets in host cells, mainly molecules with Fe–S clusters, activating or inactivating various cell functions (74, 75). Arginase modulates NO production in activated macrophages (76), what is essential in infections by trypanosomatids, as arginase activity may be involved in NOS activity impairment by competing for l-arginine and reducing macrophage microbicidal activity (77). Moreover, arginase hydrolyzes l-arginine to l-ornithine that favors parasite growth and is a precursor for the synthesis of l-glutamine, l-proline, and polyamines. Polyamines are key regulators of cell growth and differentiation (78) and essential in trypanosomatids’ antioxidant defense, which rely on trypanothione, an unusual spermidine–glutathione conjugate (Figures 2 and 3).

## Macrophage l-Arginine Metabolism Dysregulation During Trypanosomatid Infections

### NOS/Arginase Imbalance Induced by Trypanosomatids

#### Parasite Infection in the Dysregulation of the NOS/Arginase Balance

Several pathways regulate l-arginine metabolism, of which three are of interest in the context of trypanosomatids: first, in response to infection cleavage into citrulline and NO by the enzyme NOS, which is harmful since the produced NO is toxic for these parasites. Second, cleavage of arginine into ornithine and urea catalyzed by arginase, which favors trypanosome development as ornithine is a nutrient for trypanosomatids, and third, phosphorylation, in the presence of ATP, into Nω-phospho l-arginine by the arginine kinase, which allows storage of energy that can be delivered on demand, thanks to the reversibility of the reaction (arginine + ATP ↔ phospho-arginine + ADP), thus regulating energy homeostasis and contributing to trypanosomatids survival (79–81). Escaping toxic NO production requires either prevention of the activity of the NOS or a reduction in the availability of l-arginine, which may occur when several enzymes compete for this common substrate (77, 82).

Two arginase isoforms (arginase 1 and 2) have been identified in mammalian hosts so far, with a differential expression depending on tissues and cells (83, 84). Arginase activity of pathogens themselves interferes and competes in host l-arginine pathways. For instance, arginase from *Helicobacter pylori* inhibits NO production by eukaryotic cells (85). Arginase 1 and ornithine decarboxylase (ODC) are both located in the cytosol, facilitating polyamine synthesis from l-ornithine. Arginase 2 is a mitochondrial enzyme that could preferentially enhance l-proline or l-glutamate synthesis from l-ornithine because ornithine aminotransferase is also located in the mitochondria. However, it has been shown that l-proline can be converted into l-ornithine, which can be transported from the mitochondria to the cytosol (86). Both arginases 1 and 2 have been reported to regulate polyamine synthesis (87).

Trypanosomes belonging to the *brucei* group were the first parasites in which the role of host arginase induction favoring infection was evidenced (77). Considerable expansion of macrophages has been reported in the liver, spleen, and bone marrow of infected mice (88), and the presence of NOS 2 has been demonstrated in these cells. However, in infected mice, parasites proliferate in the vicinity of macrophages in the peritoneal cavity, suggesting that the efficiency of NO-dependent cytotoxicity is limited *in vivo* even though NOS 2 was active *in vitro*. Actually, a decrease in plasmatic l-arginine was measured in *Tbb-*infected mice compared to controls. An increase in arginase activity was observed in peritoneal macrophages from the first days of *Tbb* infection. Intraperitoneal NO production and NO-dependent parasite killing were restored by intraperitoneal injection of l-arginine. The early increase in arginase production in trypanosomiasis is a way for parasites to avoid the antimicrobial effect of RNI and to benefit from the larger quantities of l-ornithine that are necessary for parasite growth (77). As expected, arginase activity and arginase 1 and arginase 2 mRNA expression were demonstrated to be higher in macrophages in “trypanosusceptible”-infected BALB/c compared with those in “trypanoresistant” C57BL/6 mice (89). The high level of arginase activity in *Tbb-*infected BALB/c macrophages strongly inhibited macrophage NO production, which in turn resulted in less trypanosome killing compared with C57BL/6 macrophages. NO generation and parasite killing were restored when arginase was specifically inhibited (89). Similarly, *Tbg* field stocks isolated from patients, which did not display apparent genetic variability but marked differences in virulence (capacity to multiply inside a host) and pathogenicity (ability of producing mortality), were observed in experimental murine infections. Two strains exhibiting opposite pathogenic and virulence properties in mouse were further investigated through their host–parasite interactions. *In vitro*, bloodstream forms and corresponding secretomes from both strains induced macrophage arginase as a function of their virulence (30). Moreover, infection of mice with *T. musculi* expressing *Nippostrongylus brasiliensis* acetylcholinesterase resulted in early parasite blood clearance. It was associated with elevated NO production and lowered arginase activity, a characteristic of a modified NOS2/arginase balance (90).

Arginase 1 induction in macrophages is used by *Leishmania* species to spread inside the host, as polyamines are key elements of parasite growth (91). The proliferation of amastigotes is triggered by IL-4, IL-10, and transforming growth factor (TGF)-β *via* arginase 1 induction in macrophages leading to the generation of the polyamines required for parasite replication. On the contrary, the cytokine IL-12 plays an essential role in the initiation of adaptive responses and production of IFN-y, which is required to eliminate *Leishmania* parasites. Interestingly, it has been reported that *L. mexicana* promastigotes can activate an MAP kinase through a toll-like receptor (TLR)-4-dependent mechanism, to induce COX-2 and NOS 2 expression thereby downregulating IL-12 production (92). High splenic arginase 1 expression has been measured in an experimental model of visceral leishmaniasis caused by *L. donovani*. This detrimental activation pathway depended on the parasite-induced activation of the transcription factor STAT6, but in contrast to the previously accepted paradigm, did not require (but was amplified by) the presence of polarized Th2 cells or type 2 cytokines (93). Inhibition of arginase reduced the number of parasites and delayed disease outcome in BALB/c mice, while treatment with l-ornithine increased the susceptibility of C57BL/6 mice (94). The treatment of *L. major*-infected macrophages with Th2 cytokines (IL-4 and IL-10) or with TGF-β, which are all inducers of arginase 1, led to a proportional increase in the number of intracellular amastigotes, supporting the hypothesis that host arginase activity favors the spread of the parasite. Cell division of the parasite depends crucially on the level of l-ornithine available in the host (95).

*T. cruzi* killing by classically activated macrophages is counteracted by alternative activation, which enhances B7.2 expression, IL-10 and TGF-β production, and arginase induction (96). Macrophages are insufficiently activated in an inflammatory phenotype in response to *T. cruzi* infection, because *T. cruzi* inhibits the activation of the glycolytic pathway and the oxidative/nitrosative response in macrophage. Both arginase 1 and 2 were induced in heart tissues from *T. cruzi*-infected mice, and NOS 2 and arginase 2 were expressed by cardiomyocytes. Interestingly, heart-infiltrated CD68+ macrophages were the main cell type that expressed arginase 1 (97). Cruzipain, a major parasite antigen, was shown to induce arginase 1 expression in J774 cells, and the pretreatment of cruzipain-treated cells with N-omega-hydroxy-l-arginine (an arginase inhibitor) led to a dramatic reduction in amastigote growth. Macrophages with elevated arginase 1 activity, induced by either IL-4 or the *T. cruzi* component cruzipain, favored parasite replication and blocking arginase 1 restricted parasite growth (98).

#### Trypanosomatid Parasites ES Factors in Arginase Induction

*T. b. brucei* parasites were found to induce arginase activity in myeloid cells from non-infected mice, and activity was maintained when myeloid cells and trypanosomes were separated by a cell-retaining insert, indicating that soluble components from trypanosomes were involved. *Tbb* ES, prepared under conditions leading to no detectable trypanosome death, triggered arginase activity, but the effect was stopped by ES heat treatment. Monoclonal antibodies were raised against *Tbb* secretome and, interestingly, inhibited arginase activity induced by ES. The ES fraction, eluted after affinity chromatography, retained full arginase-inducing activity, confirming that this activity was directly targeted by an ES-specific antibody. The antibody was used to screen a cDNA expression library and identified the *Tbb* arginase-inducing protein: a kinesin heavy chain isoform (TbKHC1) (99). The secretome from TbKHC1 KO parasites did not trigger arginase activity in myeloid cells from non-infected mice, but the recombinant (r)TbKHC1 mimicked the arginase-inducing effect of secretome. Coincident with the induction of arginase activity, the secretome caused myeloid cells to express the regulatory cytokine IL-10. The arginase activity induced by ES was inhibited by a neutralizing anti-IL-10 antibody. The first peak of parasitemia in mice infected by TbKHC1 KO trypanosomes was reduced by >70% compared to wild-type parasites. A reduced TbKHC1 KO parasite load has also been observed under natural infection conditions in which infected tsetse flies were allowed to feed on mice (99).

Host mammalian macrophages are not only the main host cells but are also the main effector cells for *Leishmania* parasite killing and can be activated *via* two major pathways resulting in classical and alternative activated macrophages. *Leishmania* parasites partly activate arginase and inactivate the NO production by the host cells and enhance parasite survival *via* depletion of the NOS 2 substrate (l-arginine) and reduce NO levels. LPG, the main promastigote glycoconjugate, plays an essential role in promastigote adhesion to macrophages, rapidly fusing with lysosomes, transiently inhibiting phagosome maturation and generating a parasitophorous vacuole that maintains an acidic pH and hydrolytic activity, what provides enough time for promastigotes to differentiate into more hydrolase-resistant amastigotes (100). The replicating amastigotes produce glycoconjugates that are secreted or linked to the cell surface, such as GIPLS and proteophosphoglycan (PPG), and protect parasites from proteolytic damage (101). In parallel, it was reported that *Leishmania* parasites release increased amounts of exosomes following a shift in temperature, which strongly affect macrophage cell signaling and functions in a pro-inflammatory way to recruit neutrophils that exacerbate the pathology (42). PPG and lipophosphoglycan can facilitate the parasite survival inside the macrophages by inhibiting NOS 2 and enhancing arginase expression. During the infection, cathepsin B exported in *L. donovani* exosomes could activate TGF-β1, leading to macrophage alternative activation and enhanced parasite survival, in an arginase 1-mediated way. To regulate parasite population, *L. infantum* eukaryotic initiation factor, an exosomal protein, inhibits parasite growth through the production of TNF-α, which induces microbicidal activity by stimulating NO and reactive oxygen species (ROS) production (102). Infected sand flies regurgitate a proteophosphoglycan gel (PSG) synthesized by the parasites in the sand fly midgut, which can exacerbate cutaneous leishmaniasis. PSG was shown to rapidly recruit macrophages to the dermal site of infection and to enhance alternative activation and arginase activity of recruited macrophages, thereby increasing l-arginine catabolism and the synthesis of polyamines essential for the parasite (103).

In Chagas disease, an induction of the arginase pathway could be used by *T. cruzi* to spread inside the host (104). Interestingly, different proteins related to similar functions have been evidenced in the exoproteome of *T. cruzi*, suggesting that the invasive strategy of the parasite is based on enhanced mechanisms dedicated to interaction, invasion, and dysregulation of host target cells, especially macrophages (105). Among the proteins secreted, cruzipain, the primary secreted lysosomal peptidase in *T. cruzi*, has been shown to induce a Th2 response and to stimulate activation of the macrophage arginase metabolic pathway, associated with a decrease in macrophage NO production (98, 106). P21 is a secreted protein expressed in all the developmental stages in the *T. cruzi* lifecycle and may play an important role in parasite internalization (107). Interestingly, recombinant P21 upregulated phagocytosis of different trypanosomatids in macrophages in a CXCR4-binding-dependent manner (108) and triggered the PI3K-AKT-mTORC1 signaling pathway that has been shown to mediate polarization into M2 macrophages (109). Actually, all factors ES by *T. cruzi* appear to have convergent effects toward arginase activation to prevent aggression and promote parasite growth (110).

### Trypanosomatid Parasites’ Own l-Arginine Metabolism Enzymes

**Figure 4:**
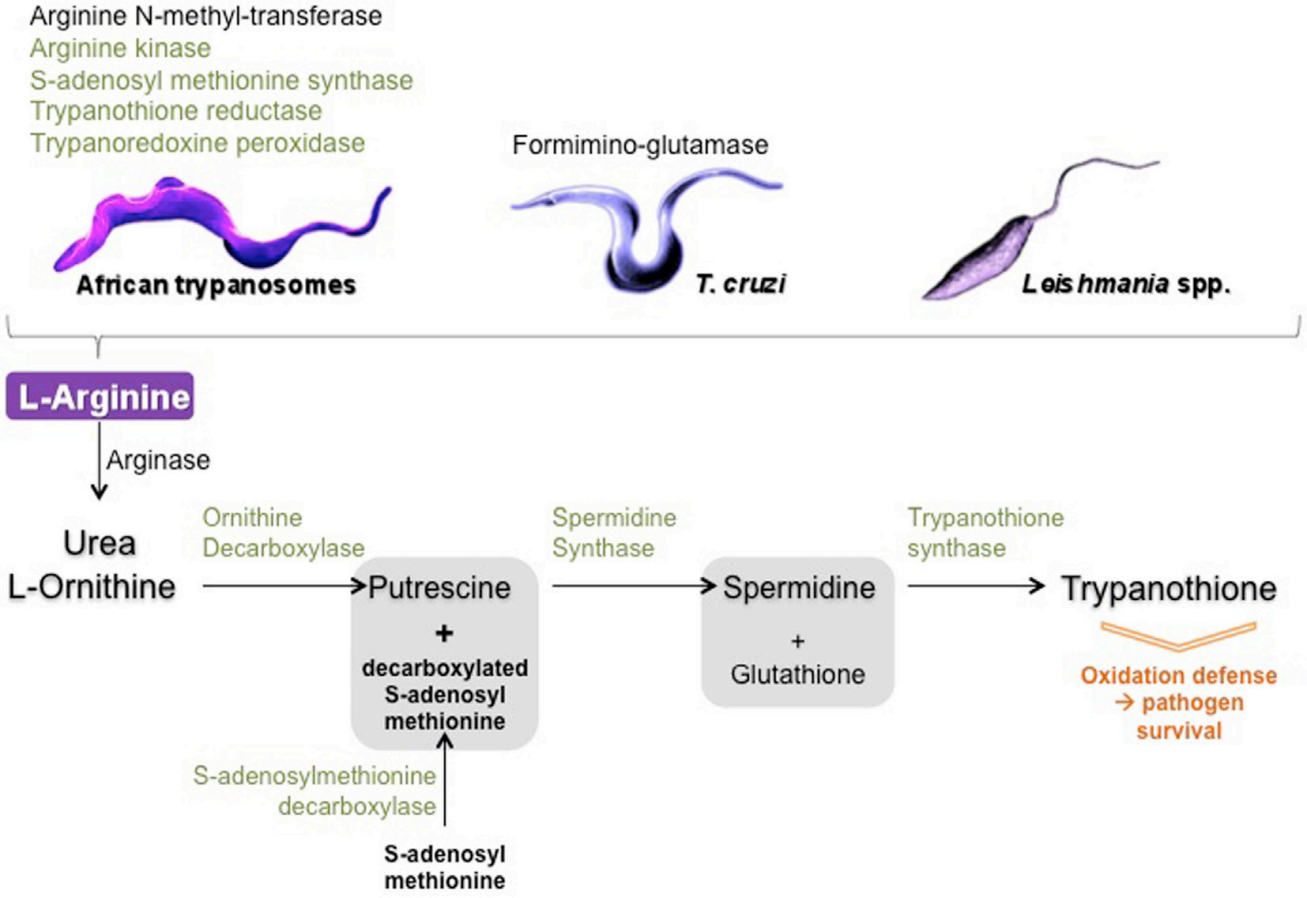
Schematic view of trypanosomatid arginase and arginine metabolism enzymes. In black: enzymes only identified in the proteome of trypanosomatids and in green: enzymes identified in the secretome of trypanosomatids.

Interestingly, in addition to ES that influence the host’s NOS/arginase balance, trypanosomatids also have several enzymes related to l-arginine metabolism, including arginase. However, *Leishmania* arginase alone is insufficient for parasite growth (111), despite it has been shown to be active in parasites isolated from patients (112) and seems to be associated with pathogenicity of the species (113). These enzymes have been shown to consume host l-arginine thereby directing host metabolism to the arginase pathway, which favors parasite development (Figure 4). Curiously, the role played by trypanosomatids’ arginase has only recently been considered to be involved in the establishment of infection in macrophages and in the immune response of the host (114). l-Arginine is an essential amino acid for *Leishmania* (115), as l-arginine deprivation or uptake determines parasite death or survival (116, 117). Induction of l-arginine transport is crucial, and to respond to l-arginine depletion in macrophage, among other transporters, *L. donovani* upregulates the expression and activity of a high affinity arginase specific transporter (118). Furthermore, in *L. amazonensis*-infected macrophages, parasite arginase downregulates NOS expression and favors *Leishmania* growth (119). Moreover, *Leishmania* parasites can modulate their own NOS-like/arginase balance (120), for instance by sensing available l-arginine and regulating its uptake (121). In *T. cruzi*, formiminoglutamase has been characterized as an arginase-like enzyme (122), and in *Leishmania* the crucial role of arginase depends on the developmental stage of the parasite (123), which adds to the complexity of modulating l-arginine metabolism by trypanosomatids. In African trypanosomes, arginase has only been identified in proteome, whereas arginine kinase has been detected as soluble and constitutive isoforms (29, 124). In addition, an arginine *N*-methyl transferase has been detected and reported to play an important biological role as it is involved in the methylation of over 800 proteins in *Tbb* (125, 126). Interestingly, arginine kinase and arginine *N*-methyl transferase genes were overexpressed in *Tbg* isolated from tsetse flies (127), as if targeting l-arginine were a metabolic key in the developmental life cycle of African trypanosomes. l-Arginine transporters were also defined as essential for trypanosomes (128). Differences in arginase subcellular locations between *Tbb* and in *T. cruzi* have been reported (129, 130), but their biological significance remains to be determined. Finally, besides arginase, two other enzymes from trypanosomatids compete with host enzymes for the same substrate, l-arginine.

Another crucial pathway deserves to be mentioned: the polyamine–trypanothione pathway, which is also connected to l-arginine metabolism and is unique to trypanosomatids (131). The biosynthetic sequence includes the following major catalyzing steps: arginase (l-arginine ⇑ urea + l-ornithine), ODC (l-ornithine ⇑ putrescine), spermidine synthase [decarboxylated S-adenosyl methionine (produced by the S-adenosylmethionine decarboxylase) + putrescine ⇑ spermidine], and as a final step, the trypanothione synthase catalyzes the biosynthesis of trypanothione from glutathione and spermidine (131–134). Trypanothione is of crucial importance as this compound, which is specific of trypanosomatids, is mainly involved in detoxifying ROS, free radicals, and, more generally, in combating various kinds of stress that occur during the parasite’s lifespan. Thus, for example, parasites lacking trypanothione reductase were shown to be avirulent and susceptible to oxidative stress (135). Of interest is the fact that most of the enzymes involved in trypanothione metabolism were identified in ES of *Tbg*: S-adenosyl-methionine synthase, spermidine synthase, trypanothione synthase-amidase, trypanothione reductase, and tryparedoxin peroxidase. The total proteome was shown to contain, in addition to the enzymes cited above, ornithine carboxylase that together with the arginase also identified in the total proteome insures the connection between the strict arginine pathway and the trypanothione pathway (29). In *T. cruzi*, some of the enzymes involved in trypanothione metabolism were also identified in the secretome (36). In *Leishmania*, enzymes secreted in the trypanothione pathways were shown to directly participate in parasite virulence and in modulating macrophage response (136).

All the abovementioned enzymes have already been described in a very large panel of reports, but only a few reported they could be ES by the parasites. Some of them, including arginase and ODC, seem to be only intracellular, but their reaction products (as well as those possible secreted by the parasite hosts—either insects or mammals) could be excreted and become the substrate of the excreted enzymes (Figure 4). How they work and their real effectiveness *in vivo* presents a large field for further investigations.

### Host Receptors in Arginase Signaling

**Figure 5:**
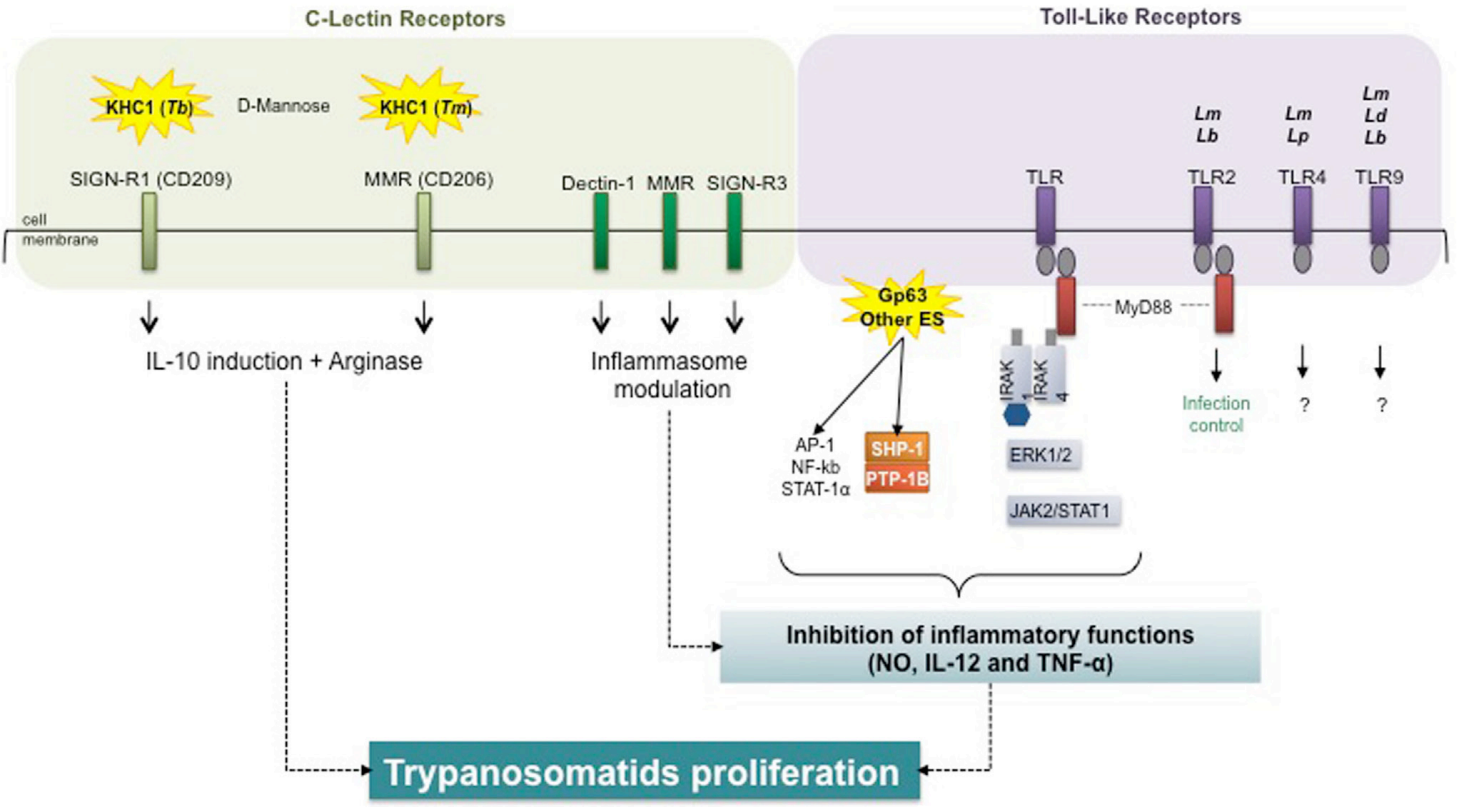
Host receptors in trypanosomatid-mediated arginase modulation. Trypanosomatids and virulence factors: *Tb, Trypanosoma brucei; Tm, T. musculi; Lm, Leishmania major*; *Lb, L. braziliensis; Lp, L. pifanoi*; *Ld, L. donovani*; Gp, glycoprotein; ES, excreted–secreted. Macrophage receptors: SIGN-R1, specific intercellular adhesion molecule grabbing non-integrin receptor 1; MMR, macrophage mannose receptor; TLR, toll-like receptor. Signal transduction molecules: MyD88, myeloid differentiation primary response 88; IRAK, interleukin-1 receptor-associated kinase; AP, activator protein; NF-κB, nuclear factor-kappa B; STAT, signal transducers and activators of transcription; SHP, Src homology region 2 domain-containing phosphatase; PTB, protein tyrosine phosphatase; ERK, extracellular signal-regulated kinase; JAK, Janus kinase (or just another kinase). Inflammatory markers: NO, nitric oxide; IL, interleukin; TNF, tumor necrosis factor.

Current research has focused on modification of host cell signaling by pathogens. For instance, C-type lectin receptors (CLRs), expressed in large quantities by DCs and macrophages, play important roles in various aspects of the immune response to pathogens (137). Upon infection, a plethora of host macrophage receptors actively respond to the invading trypanosomatids by activating several signal cascades (Figure 5).

The *in vitro* induction of arginase activity by *Tbb*, ES, and rTbKHC1 was inhibited by d-mannose (99). Parasite load and arginase activity decreased in specific intercellular adhesion molecule grabbing non-integrin receptor 1 (SIGN-R1) (CD209) KO but not in macrophage mannose receptor (MMR) KO (CD206)-infected mice. In myeloid cells from SIGN-R1 KO mice rTbKHC1 did not stimulate IL-10 and arginase 1 activity, contrary to myeloid cells from MMR KO mice. Treatment with mannose also reduced parasitemia in mice infected by *T. musculi*. However, whereas TbKHC1 facilitates *Tbb* parasitemia *via* the SIGN-R1 receptor, the MMR receptor was apparently the main target of *T. musculi* ES (138). This suggests that kinesin heavy chain-related proteins play similar roles in promoting infection in two genetically distant trypanosomes, *via* macrophage arginase induction, following distinct CLR targeting. Kinesins with close structures might act on distinct membrane receptors by recognizing related carbohydrate structures.

The initial binding and internalization of *Leishmania* promastigotes implicate the receptor-mediated classical endocytic pathway (139). This pathway involves a wide diversity of opsonic or pattern-recognition receptors, such as CR3, CR1, Fc receptors, or lectin receptors, such as the mannose fucose receptor (mannan-binding protein) and the integrin family (140, 141). The macrophage response against *L. infantum in vivo* is characterized by an M2b-like phenotype and CLR signature composed of dectin-1, MMR, and the DC-SIGN homolog SIGNR3 expression. Signals downstream from SIGNR3 shift macrophages toward a permissive state best reflected by the lower rate of parasitic proliferation in SIGNR3-deficient macrophages, suggesting that SIGNR3 modulates inflammasome activation for the benefit of the parasite (142). An important step in this immune evasion process is activation of the host protein tyrosine phosphatase SHP-1 by *Leishmania*, which directly inactivates JAK2 and Erk1/2 and contributes to the inactivation of critical macrophage inflammatory functions (e.g., NO, IL-12, and TNF-α production). SHP-1 is also involved in the inhibition of TLR-induced macrophage activation by binding to and inactivating IL-1-receptor-associated kinase 1 (143).

Toll-like receptors have been shown to impair macrophage effective immunity against intracellular pathogens through arginase 1 induction (144). TLRs were identified as determining the outcome of *L. major* (145, 146) and *L. braziliensis* (147) infections, with TLR-2 ligation and myeloid differentiation primary response 88 play an important role in infection control. Additionally, TLR-4 was demonstrated to be important in *L. major* (148, 149) and *L. pifanoi* (150) infections; TLR-9 in *L. donovani, L. major*, and *L. braziliensis* infections (151, 152), but knowledge concerning subsequent intracellular signaling is lacking. TLRs are involved in initial interactions and in downstream activation of NOS 2 and COX-2, making them key players in subsequent macrophage activation, all the more so, since TLR4 may be involved in arginase 1 induction (92).

In *T. cruzi* infection, MMR expression was upregulated in macrophages and cruzipain enhanced mannose receptor recycling, thereby favoring arginase induction and parasite survival. Moreover, receptor blockade decreased arginase activity and parasite growth in *T. cruzi*-infected mice (153).

## Concluding Remarks

Trypanosomatids insure their survival and propagation within their host by altering the signaling pathways involved in the ability of macrophages to kill pathogens or to activate the adaptive immune system. All the data presented here underline the importance of arginase induction for extra- and intracellular trypanosomatids and confirm the identity of the parasite molecules and host receptors involved. The advance in our understanding of the evasion mechanisms used by trypanosomatids enabled by these data should help to develop more efficient anti-trypanosomatids therapies in the near future. A illustrated here, the dysregulation of host l-arginine inducible metabolism by trypanosomatids ES is an effective mechanism used by the parasite to hamper host immune response and to modify host molecule production to favor parasite invasion and growth. Therefore, preventing this host metabolism dysregulation through drugs or immunization against ES active components or by blocking partner host molecules is a promising way to tackle trypanosomatid-mediated diseases.

Nevertheless, arginase triggering should be addressed with caution, as the urea cycle is essential in hosts. NOHA, a stable intermediate in NO synthesis and also an arginase inhibitor, has been shown to limit both lesion size and the parasite load in *L. major*-infected mice (94). New arginase inhibitors targeting macrophage arginase is a promising approach (154). Likewise, siRNA systems have been developed to knock down arginase 1-specific gene expression (155). Signaling is also a potential target, as inhibition of STAT3 signaling reduced arginase activity in myeloid derived suppressor cells from cancer patients (156). Blocking arginase induction, for instance by CLR-specific targeting, is another possible strategy (157, 158). On the other hand, more specific inhibition of the parasite molecules that induce host arginase activity could be an effective strategy with no side effects.

Interestingly, ES from *L. infantum* elicited a protective immune response in dogs, their natural hosts, by triggering a Th1-dominant immune response and an appropriate specific antibody response, thereby countering the parasite-induced arginase metabolism early on, and leading to the first anti-Leishmania vaccine commercially available in Europe (159–161). More recently, a secreted promastigote surface antigen, one of the main constituents and the highly immunogenic antigen of *Leishmania*, was shown to confer high levels of protection in naive dogs (162).

*Trypanosoma cruzi* secretes proteins that promote host cell invasion, and several studies have focused on the characterization of *T. cruzi* excretory–secretory antigens that are possible candidates for a vaccine. The most promising candidate appears to be the primary secreted lysosomal peptidase cruzipain, which plays vital roles in the *T. cruzi* life cycle, including triggering host arginase (163). Deleting a C-terminal domain in cruzipain led to an efficient immune response against N-terminal domain, which reduced the parasite load after a *T. cruzi* challenge (164). In addition, a new trans-sialidase-based immunogen was able to confer protection in a later *T. cruzi* challenge, by influencing populations of cells related to immune control, particularly in reducing splenic myeloid suppressor cells (165).

Like for *T. cruzi*, a sialidase-based vaccine provided partial protection in *T. b. brucei*-infected mice (107). Various approaches to vaccination against African trypanosomiasis have been investigated [reviewed in the study by LaGreca and Magez (166)]. A monoclonal antibody directed to TbKHC1 reduced arginase activity and parasite load in *T. musculi*-infected mice, and bioinformatics analysis revealed TbKHC1 homologs in other trypanosomes, including human pathogens (138). This trypanosome-specific invariant antigen is a promising candidate for a pan-trypanosome vaccine, by helping the host immune system to efficiently counter the parasite-induced arginase pathway.

The biological knowledge on how trypanosomatids and their ES factors modulate the inducible macrophage l-arginine metabolism deserves further sustained investigations to keep on prospecting for new strategies of interference in the infectious processes, whether through vaccine development or immunotherapeutic treatments.

## Author Contributions

PH, AG, RN-B, JP, SH, VR, F-AD, J-LL, PV, and RB-G defined the conception of the review, wrote the review, approved the version to be published, and agreed to be accountable for all aspects of the review.

## Conflict of Interest Statement

The authors declare that the research was conducted in the absence of any commercial or financial relationships that could be construed as a potential conflict of interest.
